# COVID-19, social determinants of transmission in the home. A population-based study

**DOI:** 10.1093/eurpub/ckae016

**Published:** 2024-02-23

**Authors:** Jesús Soriano López, Jesús Humberto Gómez Gómez, Monica Ballesta-Ruiz, Rocio Garcia-Pina, Inés Sánchez-Rodríguez, Bertha A Bonilla-Escobar, Diego Salmerón, Berta Suárez Rodríguez, Maria-Dolores Chirlaque

**Affiliations:** Murcia Region Health Department, Murcia, Spain; Teaching Unit of Preventive Medicine and Public Health, Murcia, Spain; Murcia Region Health Department, Murcia, Spain; Department of Epidemiology, Murcia, Spain; CIBER in Epidemiology and Public Health (CIBERESP), Madrid, Spain; IMIB Arrixaca, Murcia, Spain; Murcia Region Health Department, Murcia, Spain; Department of Epidemiology, Murcia, Spain; IMIB Arrixaca, Murcia, Spain; Division of Preventive Medicine and Public Health, Department of Public Health Sciences, University of Murcia, Murcia, Spain; Murcia Region Health Department, Murcia, Spain; Planning and Health Financing Department, Murcia, Spain; Murcia Region Health Department, Murcia, Spain; Department of Epidemiology, Murcia, Spain; IMIB Arrixaca, Murcia, Spain; Government of Spain Ministry of Health, Health Promotion and Equity Area, Deputy Directorate General for Health Promotion and Prevention, Directorate General for Public Madrid, Comunidad de Madrid, Madrid, Spain; TRAGSATEC, Management of Health, Food Safety and Public Health Madrid, Comunidad de Madrid, Madrid, Spain; CIBER in Epidemiology and Public Health (CIBERESP), Madrid, Spain; IMIB Arrixaca, Murcia, Spain; Department of Health & Social Sciences, University of Murcia, Murcia, Spain; CIBER in Epidemiology and Public Health (CIBERESP), Madrid, Spain; Government of Spain Ministry of Health, Spain Centre for Health Alerts and Emergencies, Directorate General of Public Health, Ministry of Health Madrid, Comunidad de Madrid, Madrid, Spain; Murcia Region Health Department, Murcia, Spain; Department of Epidemiology, Murcia, Spain; CIBER in Epidemiology and Public Health (CIBERESP), Madrid, Spain; IMIB Arrixaca, Murcia, Spain; Division of Preventive Medicine and Public Health, Department of Public Health Sciences, University of Murcia, Murcia, Spain

## Abstract

**Background:**

Studying transmission within the home is essential to understand the transmission dynamics of numerous infectious diseases. For Coronavirus Disease-2019 (COVID-19), transmission within the home constitutes the majority exposure context. The risk of infection in this setting can be quantified by the household/intra-family secondary attack rate (SAR). In the literature, there are discrepancies in these values and little information about its social determinants. The aim of this study was to investigate transmission in the home by analyzing the influence of occupational social class, country of origin and gender/sex.

**Methods:**

This was a retrospective cohort study of a population registry of cohabiting contacts with COVID-19 cases diagnosed from 15 June to 23 December 2020, in the Murcia Region. The household SAR was analyzed considering the characteristics of the primary case (sex, age, symptoms, occupational social class, country of origin and number of people in the household) and contact (age and sex) using a multilevel binary logistic regression model.

**Results:**

Among the 37 727 contacts included, the intra-family SAR was 39.1%. The contacts of confirmed primary cases in the migrant population (Africa and Latin America) had higher attack rates, even after adjusting for the other variables. Older age and female sex were independent risk factors for contracting Severe Acute Respiratory Syndrome Coronavirus 2 (SARS-CoV-2) within the home.

**Conclusion:**

There was greater intra-domiciliary transmission among immigrants, likely related to the conditions of the home and situation of social vulnerability. Women were more likely to be infected by transmission from a cohabiting infected individual.

## Introduction

Studying transmission within the home is essential to understand the infection dynamics of diseases transmitted by respiratory secretions, for example, influenza, varicella, and respiratory syncytial virus (RSV).^1^^,^^2^ Compared with other contexts (social leisure and work), the home is an exposure setting of high interpersonal contact, more defined and more easily traceable with narrow contacts. Transmission in this setting can be estimated by the household/intra-family secondary attack rate (SAR). This indicator is the proportion of secondary cases that appear among the members of a household during a period of time as a consequence of contact with a relative who presents infection (primary case). It represents a good approximation to general infectivity and may be affected by social determinants of health, such as living conditions and characteristics of the home, also linked to income level and country of origin.^3–6^ During Coronavirus Disease 2019 (COVID-19), the home was the most contagious area,^7^^,^^8^ assuming more than 50% of the confirmed cases in Spain.^7^ National and international studies on the household SAR for Severe Acute Respiratory Syndrome Coronavirus-2 (SARS-CoV-2) show very disparate figures, ranging from rates <10% to >60%.^9–24^ This was influenced by the definitions used for secondary cases and contact at home (restricted or not to cohabitants at home).^13–16^^,^^19^^,^^23^ Another key element in the analysis of these discrepancies is the different follow-up protocols used to detect secondary cases between close contacts.^13–15^^,^^19–21^ Thus, those with the highest SAR were those who performed polymerase chain reaction (PCR) tests at the end of quarantine on asymptomatic contacts, with or without a complementary serological study.^10–14^^,^^16^^,^^19^

As with other communicable diseases, differences in COVID-19 infection^25^ are associated with social determinants such as country of origin,^8^ sex,^8^^,^^26^ occupation^8^^,^^26^^,^^27^ and income.^28^ In October 2020, the Spanish Ministry of Health published an analysis of the social determinants that influenced epidemiological vulnerability to COVID-19.^6^ Regarding the in-home setting, several studies have highlighted the importance of housing conditions during the pandemic.^3^^,^^29^ There are few studies that analyze the social determinants that influence the interfamily SAR,^13^^,^^24^ and no studies have evaluated occupational social class.

The objective of this study was to investigate the influence of social determinants (country of origin, occupation and sex) on the household SAR for contacts of confirmed COVID-19 cases in the Murcia Region from June 15 (after the first wave and start of the de-escalation) to 23 December 2020 (before Christmas and the start of the vaccination campaign).

## Methods

### Study population and inclusion and exclusion criteria

This was a retrospective cohort study of the household contacts of incident cases of COVID-19 confirmed from 15 June to 23 December 2020, in the Murcia Region.

The information used was obtained from epidemiological surveys carried out for the surveillance and control of COVID-19, in which retrospective (investigating the origin of infection) and prospective (active contact search) tracing were performed. The definitions of case and close contact were consistent with the ‘Strategy for the early detection, surveillance and control of COVID-19’ released by the Ministry of Health.^30^ The surveys had uniform coverage of the entire Murcia Region, with the collection of PDIAs (active infection diagnostic test, acronim in Spanish) carried out both in public and private health systems. Surveillance was carried out in a centralized and hierarchical manner, with a systematic review of the recorded information. A procedure manual^31^ and internal communication forums were available. Epidemiologically linked cases were assigned to the same team.^31^ This structure made it possible for the contacts of confirmed cases to be assigned to a single index case (the most likely primary), minimizing the possibility that a contact would be assigned to several primary cases.

Following the national protocol,^30^ when a person presented a clinical picture compatible with COVID-19, all cohabiting people were instructed to start quarantine pending microbiological confirmation. Close contacts were followed up by both primary care and public health personnel, with successive telephone calls at variable intervals to verify compliance with measures and record symptoms. During the study period, the regional contact follow-up protocols included a second PDIA (mostly PCR) at 6–10 days from the date of last contact (DLC) with the asymptomatic contacts with the first PDIA (PCR and/or antigen test) initially negative or absent. No reinfections were reported during the study period.

Household contacts were considered only those cohabitants at the same address (who sleep there) with a primary case resident in the Murcia Region at the time of diagnosis. Resolving possible discrepancies between the primary case, the index case, and the secondary case has been a common problem in other studies^10^^,^^12^^,^^13^^,^^23^^,^^24^; therefore, secondary cases with a PDIA sample collection date during the 7 days prior to the DLC with their index case (probably not the actual primary case) were excluded, as were the rest of their cohabitants, to minimize classification error. In addition, when a person was registered on several occasions (as a primary case and/or as a contact) in a period of 24 days, the record was considered repeated. In this situation, data were cleansed based on the date of onset of symptoms (date of sampling in asymptomatic patients) for the index case, keeping only cohabiting contacts of the index case who first showed symptoms (most likely primary), excluding the rest of the records. Finally, in the remaining unique contacts, those with confirmed previous infection before 7 days of the DLC (due to their higher level of immunity) and those who were not evaluated by PDIA from the DLC until 24 days later were excluded. In the contacts included, confirmed secondary cases were considered to be those with a PDIA with a positive result recorded in that period, and cases were discarded as secondary cases in case of a negative result. A total of 98.1% of secondary cases occurred in the first 14 days after the DLC, and <0.5% occurred after the 20th day. Extending the period to 28 days from the DLC, the number of secondary cases included would have increased by 0.2%, having added 10 additional days to the 14-day incubation period.^30^

This study was approved by the ethics committee of the Virgen de la Arrixaca University General Hospital (Internal Code 2021-9-11-HCIUVA approved on 08/28/2021).

### Variables and statistical analysis

In the epidemiological surveys carried out on the confirmed cases, sociodemographic, clinical, laboratory and epidemiological variables were collected in accordance with the Ministry's strategy.^30^ From the epidemiological follow-up of close contacts, age, sex, DLC and the results and dates of PDIAs were collected.

The variable country of origin of the primary case was defined as that from which immigrants left for Spain. County of origin included the following categories: Spain (non-immigrants), Africa, Latin America, Europe/USA and Asia.

Occupation/activity was included. Primary cases with paid employment were coded and classified in accordance with the proposal prepared by the Spanish Epidemiology Society based on the International Standard Classification of Occupations^32^ designed to estimate social status based on job title (Supplementary table S1), as more fully described in another study.^8^ Category I includes directors, managers and professions requiring undergraduate degrees, and category VI corresponds to unskilled jobs. Individuals were grouped into manual workers (I, II and III) and non-manual workers (IV, V and VI).

The number of people in the household at the time of infection was calculated by adding 1 (primary case) to the number of cohabiting contacts stated by the primary case in the epidemiological survey.

The household SAR was considered the ratio between cohabiting contacts that became confirmed secondary cases of COVID-19 as a result of exposure to the primary case (excluded from the calculation) and total cohabiting contacts. The definition of confirmed case used included all microbiologically confirmed cases, in line with the Ministry’s strategy^30^ and most of the relevant published studies.^9–15^^,^^19^^,^^23^^,^^24^ The intra-family SAR was determined using characteristics of the primary case (age, sex, symptoms, occupational social class divided into non-manual workers vs. manual workers, country of origin and number of cohabitants in the home) and individual characteristics of the contact (age and sex), calculated as the quotient between the total secondary cases and close contacts evaluated within each stratum, an approach analogous to that in other studies.^10^^,^^13–19^^,^^22^

Due to the possible aggregate structure of the data between the primary cases (macro level) and their dependent secondary cases in the household (micro level), multilevel logistic regression models were carried out for the variable confirmed or discarded secondary case of the contact, verifying the hierarchical structure of the data through the intra-class correlation coefficient of a first null model that included only the identification variable of the primary case. A high intra-class correlation index is indicative that the cluster variables (primary case) explain much of the variability observed in the SAR. For the study of the household SAR, sociodemographic and epidemiological variables of the index cases and contacts were added to the null model. The final multivariate model was the one obtained through the Akaike information criterion.

Statistical analysis was performed using Stata version 13. *P* < 0.05 was considered significant.

## Results

**Figure 1:**
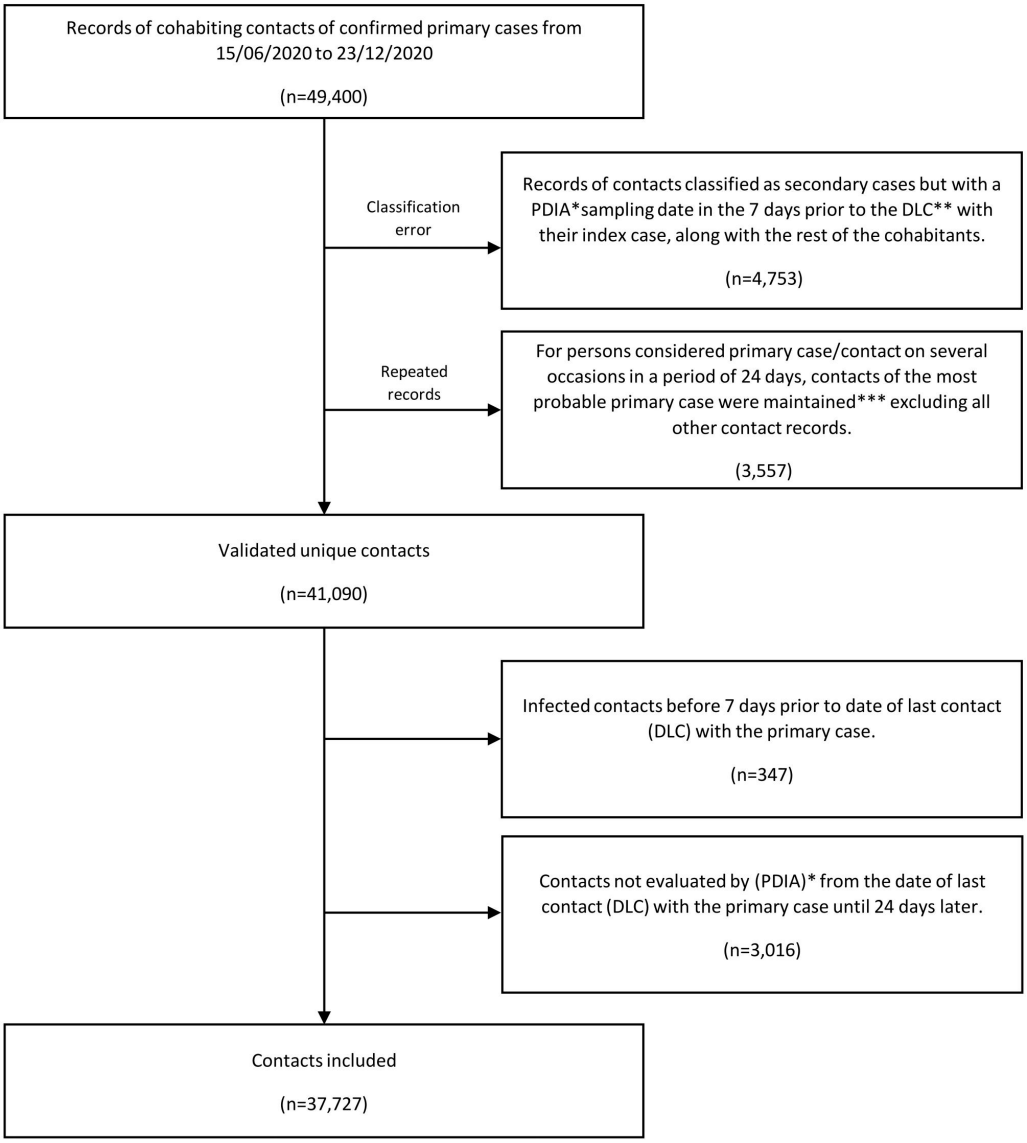
COVID-19. Inclusion and exclusion criteria for household contacts of confirmed primary cases. Murcia Region 2020 (June 15 to December 23). *PDIA Active infection diagnostic test (PCR or antigen). **DLC: date of last contact. ***Most probable primary: individual who first experienced symptoms (date of diagnosis if not)

Of the 49 400 records of cohabitating contacts with diagnosed cases in the Murcia Region from 15 June to 23 December 2020 (figure 1), secondary cases with a sampling date in the 7 days prior to the DLC with their index case along with their cohabitants (4753 records) were excluded. In addition, repeated records were purged, excluding 3557 additional records. Of the remaining 41 090 unique contacts, those with previous infection 7 days after the last contact (347) and not evaluated by PDIA (PCR or antigen test) from the DLC until 24 days later (3016) were excluded. The percentage of close contacts not evaluated by PDIA was similar between contacts of confirmed cases who emigrated from Africa (9.4%) and Latin America (6.6%) and contacts of confirmed cases who were non-migrants (7.2%). As a result, 37 727 close contacts of 16 244 primary cases/households were included.

There were differences (*P* < 0.05) in the characteristics of close cohabiting contacts (age and sex) and their primary cases (age, sex, symptoms, occupation and number of cohabitants) depending on the country of origin of the cases (table 1). The greatest discrepancies between immigrants and non-immigrants (Spain in the tables) were observed for the age and occupation of the primary cases, with a higher proportion of people under 65 years of age and with occupations defined as manual among primary cases confirmed in the migrant population.

**Table 1:** COVID-19: characteristics of the primary cases according to their country of origin and of their household contacts according to the country of origin of their primary/index case: Murcia Region 2020 (15 June to 23 December)

				Country of origin											
				Spain		Africa		Latin America		Europe, USA		Asia		Total (includes unknown country)	
				*N*	(%)	*N*	(%)	*N*	(%)	*N*	(%)	*N*	(%)	*N*	(%)
Primary case variables	Sex														
	Women			6301	(52.8)	532	(47.8)	1613	(59.8)	190	(58.8)	18	(35.3)	8727	(53.7)
	Age, years														
	0–14			1183	(9.9)	45	(4.0)	32	(1.2)	6	(1.9)	3	(5.9)	1271	(7.8)
	15–34			3772	(31.6)	295	(26.5)	931	(34.5)	86	(26.6)	14	(27.5)	5138	(31.6)
	35–64			5565	(46.6)	727	(65.3)	1689	(62.6)	219	(67.8)	33	(64.7)	8309	(51.2)
	≥65			1417	(11.9)	46	(4.1)	46	(1.7)	12	(3.7)	1	(2.0)	1526	(9.4)
	Symptoms														
	Symptomatic			9420	(80.0)	716	(67.9)	2099	(79.4)	263	(82.4)	37	(75.5)	12619	(79.1)
	Activity/occupational social class														
	Homemaker			339	(2.8)	88	(7.9)	33	(1.2)	8	(2.5)	4	(7.8)	472	(2.9)
	Students			2309	(19.3)	75	(6.7)	100	(3.7)	23	(7.1)	6	(11.8)	2518	(15.5)
	Unemployed			1196	(10.0)	262	(23.5)	391	(14.5)	38	(11.8)	13	(25.5)	1921	(11.8)
	Paid employees^a^			6324	(53.0)	587	(52.7)	2064	(76.5)	230	(71.2)	26	(51.0)	9309	(57.3)
	Occupational Social Class	Non-manual	I	856	(13.5)	3	(0.5)	26	(1.3)	13	(5.7)	0	(0.0)	902	(9,7)
			II	678	(10.7)	8	(1.4)	11	(0.5)	14	(6.1)	1	(3.8)	712	(7,6)
			III	1179	(18.6)	10	(1.7)	46	(2.2)	30	(13.0)	2	(7.7)	1276	(13,7)
		Manual	IV	639	(10.1)	26	(4.4)	105	(5.1)	25	(10.9)	0	(0.0)	796	(8,6)
			V	2135	(33.8)	170	(29.0)	822	(39.8)	98	(42.6)	3	(11.5)	3256	(35,0)
			VI	837	(13.2)	370	(63.0)	1054	(51.1)	50	(21.7)	20	(76.9)	2367	(25,4)
	Pensioners			1507	(12.6)	40	(3.6)	36	(1.3)	17	(5.3)	0	(0.0)	1604	(9.9)
	Unidentified^b^			262	(2.2)	61	(5.5)	74	(2.7)	7	(2.2)	2	(3.9)	420	(2.6)
	No. of people at home														
	2–3 people			6943	(58.2)	433	(38.9)	1460	(54.1)	214	(66.3)	20	(39.2)	9137	(56.2)
	4–6 people			4731	(39.6)	604	(54.3)	1140	(42.3)	102	(31.6)	28	(54.9)	6653	(41.0)
	>6 people			263	(2.2)	76	(6.8)	98	(3.6)	7	(2.2)	3	(5.9)	454	(2.8)
	Total			11937	(100.0)	1113	(100.0)	2698	(100.0)	323	(100.0)	51	(100.0)	16244	(100.0)
Close contact variables	Sex														
	Women			13706	(51.2)	1494	(45.8)	3201	(48.9)	331	(50.2)	62	(42.2)	18925	(50.2)
	Age, years														
	0–14			6120	(22.8)	1318	(40.4)	1911	(29.2)	166	(25.2)	45	(30.6)	9662	(25.6)
	15–34			7016	(26.2)	916	(28.0)	2233	(34.1)	194	(29.4)	48	(32.7)	10490	(27.8)
	35–64			11304	(42.2)	985	(30.2)	2252	(34.4)	261	(39.5)	51	(34.7)	14953	(39.6)
	≥65			2369	(8.8)	47	(1.4)	157	(2.4)	39	(5.9)	3	(2.0)	2622	(6.9)
	Total			26809	(100.0)	3266	(100.0)	6553	(100.0)	660	(100.0)	147	(100.0)	37727	(100.0)
Contacts not evaluated by PDIA (not included in table)				2087	(7.2)	340	(9.4)	475	(6.6)	51	(7.1)	27	(15.4)	3016	(7.3)

Notes: A total of 293 primary cases did not have information on symptoms, and 122 did not have information about country of origin. For five close cohabiting contacts, no sex information was available.

a Paid employees is broken down by occupational social class, indicating a percentage with respect to the total number of paid employees.

b Occupation not coded because the field is empty or not enough information was available.

The SAR was 39.1% [95% confidence interval (CI): 38.7–39.6%] as seen in table 2, which shows this indicator by characteristics of the primary case. The SAR was higher among contacts whose primary case was an immigrant from Africa (51.6%) and Latin America (50.8%) than among those infected by non-immigrant cohabitants (34.8%) or with Europe or the USA as the country of origin (35.0%). Although higher rates were observed in households with jobs classified as manual (41.7%) vs. non-manual (35.3%), this difference disappeared when the data were by grouped country of origin. In non-immigrant primary case contacts, the SAR was higher in households with two to three members (38.5%) than in those with four to six people (32.8%) and over six people (30.2%). Among immigrants from Africa and Latin America, the highest rates occurred in households with six or more people (57.6% and 53.5%, respectively).

**Table 2:** COVID-19: household SAR and 95% confidence interval by characteristics of the primary case: Murcia Region 2020 (15 June to 23 December)

	Spain		Africa		Latin America		Europe, USA		Asia		Total (includes unknown country)	
	*N* ^a^	SAR^a^	*N*	SAR	*N*	SAR	*N*	SAR	*N*	SAR	*N*	SAR
Symptoms												
Asymptomatic	1089/	20.4% (19.3–21.5%)	403/	42.2% (39.1–45.4%)	428/	33.9% (31.3–36.5%)	25/	22.1% (14.5–29.8%)	14/	40.0% (23.8–56.2%)	1980/	25.4% (24.5–26.4%)
	5336		954		1264		113		35		7783	
Symptomatic	8138/	38.5% (37.9–39.2%)	1236/	56.6% (54.5–58.6%)	28 43/	55.0% (53.6–56.3%)	203/	37.5% (33.4–41.6%)	57/	53.3% (43.8–62.7%)	12581/	42.9% (42.3–43.5%)
	21 125		2185		5171		541		107		29 322	
Sex												
Men	4323/	33.9% (33.1–34.7%)	926/	53.0% (50.7–55.4%)	1446/	52.9% (51.0–54.7%)	93/	32.5% (27.1–37.9%)	56/	55.4% (45.8–65.1%)	6890/	38.9% (38.2–39.6%)
	12 747		1746		2736		286		101		17 721	
Women	4995/	35.5% (34.7–36.3%)	758/	49.9% (47.4–52.4%)	1885/	49.4% (47.8–51.0%)	138/	36.9% (32.0–41.8%)	17/	37.0% (23.0–67.7%)	7879/	39.4% (38.7–40.1%)
	14 062		1520		3817		374		46		20 006	
Age, years												
0–14	1015/	30.1% (28.6–31.7%)	52/	37.1% (29.2–45.1%)	44/	56.4% (45.4–67.4%)	3/	20.0% (4.3–48.9%)	6/	40.0% (16.3–64.7%)	1122/	31.0% (29.5–32.5%)
	3368		140		78		15		15		3621	
15–34	2411/	28.5% (27.5–29.4%)	395/	47.1% (43.8–50.5%)	1030/	44.5% (42.5–46.6%)	54/	30.0% (23.3–36.7%)	14/	38.9% (23.0–54.8%)	3942/	33.0% (32.2–33.9%)
	8468		838		2313		180		36		11 935	
35–64	4837/	38.7% (37.9–39.6%)	1143/	53.2% (51.1–55.3%)	2195/	54.0% (52.5–55.5%)	163/	36.7% (32.2–41.2%)	51/	54.3% (44.2–64.3%)	8478/	43.6% (42.9–44.3%)
	12 497		2148		4066		444		94		19 432	
≥65	1055/	42.6% (40.7–44.6%)	94/	67.1% (59.4–74.9%)	62/	64.6% (55.0–74.2%)	11/	52.4% (28.6–76.1%)	2/	100% (-)	1227/	44.8% (42.9–46.7%)
	2476		140		96		21		2		2739	
Activity/occupational social class												
Non-manual workers	2033/	34.8% (33.6–36.0%)	30/	52.6% (39.7–65.6%)	97/	49.7% (42.7–56.8%)	31/	28.7% (20.2–37.2%)	6/	75.0% (34.9–92.8%)	2201/	35.3% (34.1–36.5%)
	5846		57		195		108		8		6238	
Workers manuals	2801/	35.2% (34.1–36.2%)	773/	48.6% (46.2–51.1%)	2434/	50.1% (48.7–51.6%)	128/	36.7% (31.6–41.7%)	45/	58.4% (47.4–69.4%)	6256/	41.7% (40.9–42.5%)
	7965		1589		4854		349		77		14 997	
Rest of cases	4484/	34.5% (33.7–35.3%)	881/	54.4% (52.0–56.8%)	800/	53.2% (50.7–55.7%)	72/	35.5% (28.9–42.0%)	22/	35.5% (23.6–47.4%)	6312/	38.3% (37.5–39.0%)
	12 998		1620		1504		203		62		16 492	
Number of people at home												
2–3 people	3850/	38.5% (37.5–39.4%)	327/	51.5% (47.6–55.4%)	1053/	50.2% (48.1–52.4%)	105/	34.8% (29.4–40.1%)	13/	37.1% (21.1–53.2%)	5390/	40.9% (40.1–41.8%)
	10 006		635		2097		302		35		13 169	
4–6 people	4934/	32.8% (32.1–33.6%)	1084/	50.3% (48.1–52.4%)	1946/	50.7% (49.1–52.3%)	116/	36.8% (31.5–42.2%)	49/	52.1% (42.0–62.2%)	8201/	38.0% (37.3–38.6%)
	15 035		2157		3836		315		94		21 588	
>6 people	534/	30.2% (28.1–32.3%)	273/	57.6% (53.1–62.0%)	332/	53.5% (49.6–57.5%)	10/	23.3% (10.6–35.9%)	11/	61.1% (35.7–82.7%)	1178/	39.7% (37.9–41.4%)
	1768		474		620		43		18		2970	
Total:	9318/	34.8% (34.2–35.3%)	1684/	51.6% (49.8–53.3%)	3331/	50.8% (49.6–52.0%)	231/	35.0% (31.4–38.6%)	73/	49.7% (41.6–57.7%)	14 769/	39.1% (38.7–39.6%)
	26 809		3266		6553		660		147		37 727	

a SAR: cohabiting contacts that become secondary confirmed cases of COVID-19/total cohabiting contacts. The quotient is displayed.

In the multilevel logistic regression model (table 3) conducted to predict which household contacts became confirmed secondary cases of COVID-19 based on variables of the primary case (grouping variable) or of the contact itself, the intra-class correlation index was 0.686 (95% CI: 0.670–0.702). Greater risk was observed for cohabitants of primary cases >65 years and symptomatic. Primary cases <15 years of age were more contagious to their cohabitants than were those between 15 and 34 years of age. Controlling for the other factors, transmission was higher in the homes of primary cases who migrated from Africa, Latin America and Asia. A household number of two to three was associated with a higher SAR than that observed in larger households. By including this variable in the model, none of the directions of the associations found between the other variables and the SAR changed, nor was the magnitude or statistical significance altered substantially. The occupational social class of the primary case (grouped into manual vs. non-manual) was not significantly associated with the SAR. A higher SAR was observed among the contacts of primary cases whose occupation was a homemaker. Older and female contacts were more contagious.

**Table 3:** COVID-19: multivariate multilevel logistic regression model to study the household SAR by the characteristics of the primary cases and of the contacts: Murcia Region 2020 (15 June to 23 December)

	Odds ratio	(95% CI)	*P*-value
Variables of the primary case (macro/group level)			
Sex			
Man (ref.)			
Female	1.11	(1.00–1.24)	0.044
Symptoms			
Asymptomatic (ref.)			
Symptomatic	4.79	(4.19–5.48)	<0.001
Age, years			
<15 (ref.)			
15–24	0.74	(0.60–0.92)	0.007
25–64	1.65	(1.33–2.05)	<0.001
≥65	2.01	(1.55–2.61)	<0.001
Country of origin			
Spain (ref.)			
Africa	4.44	(3.62–5.44)	<0.001
Latin America.	3.54	(3.05–4.11)	<0.001
Europe, USA	0.87	(0.60–1.26)	0.463
Asia	2.74	(1.16–6.48)	0.022
Activity/occupational social class			
Non-manual workers (ref.)			
Manual workers	1.11	(0.95–1.29)	0.183
Homemaker	1.43	(1.04–1.98)	0.029
Rest of primary cases	1.31	(1.11–1.54)	<0.001
Number of people at home			
2–3 people (ref.)			
4–6 people	0.75	(0.68–0.84)	<0.001
>6 people	0.67	(0.52–0.87)	0.003
Close contact variables (micro/individual level)			
Sex			
Man (ref.)			
Female	1.30	(1.22–1.39)	<0.001
Age, years			
<15 (ref.)			
15–24	1.14	(1.04–1.25)	0.007
25–64	1.28	(1.18–1.40)	<0.001
≥65	1.80	(1.52–2.12)	<0.001

Notes: Dependent variable: confirmed/discarded secondary COVID-19 case. Independent variables of the primary case/household level/macro level: sex, age, symptoms, country of origin and number of people in the household. Independent variables of the contact/micro level: sex and age.

## Discussion

In the Murcia Region, the household SAR for SARS-CoV-2 in the period prior to vaccination was conditioned by social determinants. It was higher among the contacts of immigrants from Africa, Asia and Latin America. Being a woman was associated with a greater possibility of contracting and transmitting the infection at home.

In the literature, there are discrepancies in the values reported for the household SAR for SARS-CoV-2.^9–22^ For example, although they are not comparable studies, the National Seroprevalence Study of SARS-CoV-2 Infection (ENECOVID) at the end of 2020 reported a seroprevalence of 36.2% among people who reported having previously been in contact with a positive case among the people they live with,^33^ a finding similar to that obtained in a seroprevalence study in Brazil (35%).^34^ In Navarra,^19^ from January to April 2021, the intra-familial attack rate was 50.2% among unvaccinated household contacts. In a study conducted in 2020 with a sample of 187 Dutch contacts^12^ with meticulous follow-up (including the collection of exudates and serological tests), the intra-familial SAR was 43%. Another similar study conducted in Norway reported a SAR of 45%.^10^

The higher SAR observed among contacts of symptomatic cases and those older than 65 years are consistent with the results reported in other studies.^9^^,^^12^^,^^13^ The fact that individuals with COVID-19 who are between the ages of 15 and 34 years are less contagious to their cohabitants than are those <15 years, as reported in several studies,^13^^,^^17^^,^^18^^,^^23^ may be related to the care required by the latter.

In this study, there was greater transmission among the household contacts of immigrants, a finding that can probably be explained by the inequalities in the conditions of the home that made it difficult to comply with the measures aimed at protecting cohabitants from infection. Likewise, several studies describe the influence of the precariousness of homes, without access to water or laundry and with greater difficulty social distancing.^5^^,^^6^

This probably amplified the differences in incidence^8^ caused by other inequities (employment, income, neighbourhood, etc.). A 2020 study conducted in California,^13^ in which 382 contacts were followed up with PCR and serology, reported a SAR of 80% in the homes of primary cases of Hispanic origin (92.7% of the sample) compared with 47.4% in non-Hispanic homes, even after adjusting for sex and age of the secondary case, household size and geography compared with 47.4% in non-Hispanic homes. The rate was significantly affected by household income. In a large population study conducted in Norway from August 2020 to May 2021, the SAR was higher in the homes of immigrants (32%) than in the homes of non-immigrants (20%)^24^ even after adjusting for sex and age of the secondary case, household size and geography.

Analogous to the flu,^2^ among the contacts in households with three or fewer members, the SAR was higher than in households with four to six members, a finding that coincides with the results of other studies.^10^^,^^17^^,^^18^^,^^22^^,^^24^ A plausible explanation may be that a greater number of people leads to more social interactions shared with the primary case and that in smaller families, the type of contact is more intimate and greater between spouses^22^ than between siblings, grandparents, etc. The analysis of the housing conditions (surface area per person, number of people per bathroom, number of bedrooms, ventilation, etc.) could be useful in the study of these differences. Housing overcrowding has been associated with COVID-19 risk of infection.^35^ In a study conducted on the 326 household contacts of 89 Spanish health workers during the pre-vaccination period, it was observed that when the index case used a single room, it had a protective effect.^36^ Another Spanish study found a higher likelihood of one or more secondary cases arising from a primary case in households where shared rooms or difficulties in maintaining ambient ventilation were present.^37^

Older contacts were more susceptible to becoming infected, as also reported in the previous studies.^9^^,^^20^ In the present study, female contacts presented a higher risk of infection when adjusting for the other variables, a finding that can be explained by the social-caregiver role of women at home, supporting most of the care tasks.^38^

Greater intra-household transmission was observed among the contacts of primary cases without paid employment (including students, retirees and unemployed people) than among those with paid employment, as also observed in another study,^16^ probably because of the longer time spent at home. The highest rates were observed among cohabitants of people whose activity was homemaker, a result that is likely explained by their central role within the home.

The study did not find a significant influence of the occupational social class of the primary case on the household SAR when adjusting for the other variables. This may be due to several factors. Social characteristics of the primary case, such as occupational social class, may not be fully representative of the other household members (the employment of the other cohabitants was not considered); therefore, their true relationship with the SAR may have been biased towards nullity. The European Health Survey in Spain bases the occupational social class of a household on the employment status of one person, defined as the “reference person,” which has similarities with our study. Additionally, income was not considered (neither the individual nor the rest of the family unit). A Colombian study^39^ found no relationship between the SAR and socioeconomic level. Regarding the influence of occupation, it was divided into categories such as healthcare, police/military/firefighter, construction, etc. It only found a higher SAR among close contacts of primary cases with informal employment or looking for a job, align with our study where the SAR was higher in contacts of cases without paid employment. Even it does not strictly evaluate the household SAR, 81.0% (3949) of the close contacts were from households and this variable was considered in the analysis.

Another limitation, highlighted in several studies,^10^^,^^12^^,^^13^^,^^24^ is the absence of complete coincidence between the primary case and the index case. Specific protocols^30^ and exclusion criteria have been applied to minimize this potential problem. Although contacts with confirmed previous infection were excluded, some of those included may been infected but not diagnosed microbiologically prior to the DLC, a situation that was especially important in the first wave of the pandemic, as demonstrated by the ENE-COVID study.^33^ The lower impact of COVID-19 in the Murcia Region during the first wave compared with that in other autonomous communities^33^ and the wide coverage of epidemiological surveillance during the study period minimized this potential limitation. The primary purpose epidemiological surveillance population-based registry was monitoring of the pandemic, and as a consequence, information pertaining to clinical characteristics, individual income or housing conditions was not captured. While some aggregated information is available through national surveys,^40^ but it was noted these data were outdated and did not meet the required disaggregation level to include them in the models. Information about housing surface is available in the public land registry (cadastre), but it was not feasible to link it with our data.

The main strength of this study is the capacity of the epidemiological registry to reflect the community transmission of COVID-19, as shown by its good agreement with the results of the ENE-COVID study.^33^ From the third wave (8–22 June 2020) to the fourth wave (16–29 October 2020), approximately 3.5% of residents in the Murcia Region went from being IgG− to IgG+. This translates into an estimated 52 500 cases, a figure similar to those registered in that period.^7^ The protocol of contact follow-up has great importance in the interpretation of the results.^33^ In addition, the most vulnerable immigrant population frequently does not have a fixed address; therefore, the cohabitants declared in the period of transmissibility were considered,^6^ eliminating the necessity to turn to census sources for their identification, a process often hindered by updating issues. Notably, most of the close contacts with initial negative/absent PDIA underwent a PDIA (usually PCR) 5–10 days after the DLC either by follow-up protocol or because of developing symptoms, thus reducing the percentage of potential undiagnosed cases. As the study was carried out prior to the start of the vaccination campaign, the influence of the variables studied on the SAR was not conditioned by differences in vaccination coverage according to age, occupation or different variants of the virus (it was the native variant throughout the study period).

A higher household SAR was found among the contacts of immigrants and female contacts. The results obtained provide valuable information regarding the influence of social determinants on the transmission of respiratory diseases. It is necessary to include social conditions in epidemiological surveys and in the variables collected in an automated way through digital health histories to enable epidemiological surveillance with a focus on equity and the design of effective prevention and control measures. Sex and country of origin, along with other axes of inequality, should be included in studies that evaluate the household SAR as well as vaccine effectiveness studies and mathematical models that study both the different variants of SARS-CoV-2 and other communicable diseases, such as influenza and RSV.

## Supplementary Material

ckae016_Supplementary_Data

## Data Availability

The data underlying this article will be shared on reasonable request to the corresponding author. Key points Intra-domiciliary transmission was notably higher among immigrants, with females being a risk factor for infection at home. Younger people infected with SARS-CoV-2 were more likely to transmit the virus to their cohabitants than were middle-aged people. The results highlight the importance of social determinants in the transmission of respiratory diseases. Sex and country of origin, along with other axes of inequality, should be included in epidemiological surveys and in studies that evaluate the household secondary attack rate, for example, vaccine effectiveness studies and mathematical models on the transmission of new variants of SARS-CoV-2 and other infectious diseases such as influenza and respiratory syncytial virus.
