# Supplementary material for: COVID-19, social determinants of transmission in the home. A population-based study
Source: Eur J Public Health. 2024 Feb 23;34(3):427–34. doi: 10.1093/eurpub/ckae016 (PMC11161145; doi:10.1093/eurpub/ckae016)
Supplement: ckae016_Supplementary_Data [file ckae016_supplementary_data.pdf]

Supplementary appendix

Table A: Neo-Weberian social class proposals from the National Classification of Occupations 2011

	6 categories grouped (used in Table 1)	2 categories grouped (used in Tables 2 and 3)
**I**	Directors and managers of establishments with 10 or more employees and professionals traditionally associated with university degrees	Nonmanual workers
**II**	Directors and managers of establishments with less than 10 workers, professionals traditionally associated with university degrees and other technical support professionals: athletes and artists	
**III**	Intermediate occupations and self-employed workers	
**IV**	Supervisors and workers in skilled and semiskilled technical occupations	Manual workers
**V**	Skilled workers in the primary sector and other semiskilled workers	
**VI**	Unskilled workers	

Adapted from Domingo-Salvany A, Bacigalupe A, Carrasco JM, Espelt A, Ferrando J, and Borrell C. Group of Social Determinants of the Spanish Society of Epidemiology. Proposals for social class classification based on the Spanish National Classification of Occupations 2011(32)
